# United states department of defense (DoD) real property repair, alterations, maintenance, and construction project contract data: 2009–2020.

**DOI:** 10.1016/j.dib.2020.106128

**Published:** 2020-08-05

**Authors:** Tyler Stout, Adam Teston, Brent Langhals, Justin Delorit, Carlton Hendrix, Steven Schuldt

**Affiliations:** a Department of Systems Engineering and Management, Air Force Institute of Technology, Wright-Patterson AFB, OH 45433, United States; b Technical Services Division, Facility Engineering Directorate, Air Force Civil Engineer Center, JBSA-Lackland, TX 78236, United States

**Keywords:** Construction, Cost, Schedule, Overrun, Delay, Growth, Data, Contract, Department of defense

## Abstract

Nearly one-half of all construction projects exceed planned costs and schedule, globally [1]. Owners and construction managers can analyze historical project performance data to inform cost and schedule overrun risk-reduction strategies. Though, the majority of open-source project datasets are limited by the number of projects, data dimensionality, and location. A significant global customer of the construction industry, the Department of Defense (DoD) maintains a vast database of historical project data that can be used to determine the sources and magnitude of construction schedule and cost overruns for many continental and international locations. The selection of data provided by the authors is a subset of the U.S. Federal Procurement Data System-Next Generation (FPDS-NG), which stores contractual obligations made by the U.S. Federal Government [2]. The data comprises more than ten fiscal years (1 Oct 2009 – 04 June 2020) of construction contract attributes that will enable researchers to investigate spatiotemporal schedule and cost performance by, but not limited to: contract type, construction type, delivery method, award date, and award value. To the knowledge of the authors, this is the most extensive open-source dataset of its kind, as it provides access to the contract data of 132,662 uniquely identified construction projects totaling $865 billion. Because the DoD's facilities and infrastructure construction requirements and use of private construction firms are congruent with the remainder of the public sector and the private sector, results obtained from analyses of this dataset may be appropriate for broader application.

**Specifications Table**

**Table:** 

**Subject**	Engineering (General)
**Specific subject area**	The data within are 10-plus (9 additional months of 2020 contract data) fiscal years’ repair, alterations, maintenance, and construction project contract attributes, that represent an annual multi-billion-dollar effort by the U.S. Federal Government to ensure the continued use and functionality of DoD facilities (also known as ‘real property’). These data may be used to better predict costs and durations in nearly all sectors of construction for the U.S. Federal Government. Furthermore, the data could be used to provide quantifiable performance metrics on the ability of the DoD to execute various project types.
**Type of data**	Table
**How data were acquired**	Data were acquired through the Federal Procurement Data System - Next Generation (FPDS-NG or FPDS). The FPDS-NG offers public users access to the spending patterns of the Federal government. The FPDS houses all contract actions of the Federal Government, beyond construction. Filters were applied to limit the results to just construction projects funded by the DoD.
**Data format**	Raw
**Parameters for data collection**	Access FPDS-NG website and create an ad hoc report filtering the contract data by: 1. Date Signed 2. Contracting Department Name 3. Product Service or Code
**Description of data collection (600 max characters)**	Government agencies are responsible for collecting and reporting data on federal procurements through the Federal Procurement Data System–Next Generation (FPDS-NG). Contracting Officers (COs) must submit complete reports on all contract actions, as required by the Federal Acquisition Regulation (FAR) [3]. Any contract with an estimated value greater than $10,000 must be reported using FPDS-NG [4]. FPDS-NG is the sole location for all contractual and procurement obligations made by the U.S. Federal Government.
**Data source location**	Institution: Federal Procurement Data System - Next Generation (FPDS-NG)
**Data accessibility**	Repository name: Mendeley Data Data identification number: DOI: 10.17632/yk4s7pdsvk.1 http://dx.doi.org/10.17632/yk4s7pdsvk.1 Direct URL to data: https://data.mendeley.com/datasets/yk4s7pdsvk/1

## 1 Value of the data

• These data contain 132,662 construction projects, spanning 10-plus years, and account for $856 billion in DoD spending. These data are categorically diverse; they contain many types of projects, including but not limited to, roads, runways, administrative facilities, communications work, mechanical renovation, and demolition.
• Statistical analyses may be performed by researchers participating in construction auditing, cost estimating, planning, or programming.
• These data may identify trends and relationships in construction contract information at and between geographic locations, construction sectors, contract types, contracting agents, project costs, project durations, and modification frequency.
• Current literature focuses on a comparatively small sample size when empirically analyzing construction contract data. To the author's knowledge, this is the most extensive set of construction contract data from a single source.
• These data can also be used to track historical spending on construction projects within the U.S. DoD. These data could prove useful in creating forecasting models on construction cost fluctuations or even be used to calibrate project costs and schedules based on their type.

## 2 Data description

The data were compiled from the FPDS-NG website using specific querying to obtain all real property repair, alterations, maintenance, and construction projects executed by the U.S. DoD from 2009 to 2020. These data represent 132,652 construction projects for which the U.S. DoD contracted outside entities to complete necessary maintenance, repairs, alterations, and modernization of U.S. DoD real property.

These U.S. DoD construction projects range from hangar and runway repairs to modernization projects for office space. Many of the projects completed on U.S. DoD installations can also be found in the public or private sectors of the construction industry.

Funding of U.S. DoD construction projects varies from year to year, much like other public and private entities. This variability in funding is based on factors outside of the control of the U.S. DoD and, therefore, requires these expenditures to be on-target with regard to planned cost and schedule. The effects of deviation from these planned attributes, for any project, can be far-reaching. Projects exceeding planned cost and schedule can result in deferred or canceled facility maintenance, repair or construction initiatives elsewhere in the DoD's portfolio, both in the current and future years. To ensure the capability and mission readiness of the U.S. DoD (of which the U.S. military is a part), the facilities it operates must be maintained to meet the users’ needs.

To mitigate these deferments, possible project cancellations, and in order to meet the needs of the facility occupants, these data can be used to identify key factors associated with cost and schedule deviations. Once isolated, these factors can be used to mitigate future cost or schedule overruns associated with public and private construction, as well as U.S. DoD construction projects.

## 3 Experimental design, materials, and methods

As mentioned previously, the data were pulled from FPDS-NG using several progressive filters. The filters used are listed below:

1 “Contracting Department Name” showing only “DEPT OF DEFENSE”
2 “Product Service Code” similar to “Y1” for “Construction of Structures and Facilities”
3 “Product Service Code” similar to “Z1” for “Maintenance of Real Property”
4 “Product Service Code” similar to “Z2” for “Repair of Alterations of Real Property”
5 “Date Signed only show values between” with dates “10/01/XXXX” and 09/31/XXXX” based on the fiscal year (e.g., 10/01/2017 and 09/31/2018 for fiscal year 2018)
6 “Treasury Account Symbol Main Account Code” showing only “3400″, “3300″, “3307″, “3404″, “1205″, “1206″, “1804″, “1805″, “1106″, “1107″, “2020″, “2022″, “2050″, “2051″, “3122″, “3123″,”'3134”, “3135.”
7 Each Product Service Code was used for every fiscal year while keeping the Contracting Department Name consistently limited to the Department of Defense. In doing so, at least three spreadsheets were produced for each fiscal year from 2009 through the first 6 months 2020. The database output was limited to CSV files containing 30,000 or fewer lines that, in some cases, necessitated the production of additional files based on a given PCS and fiscal year.

A complete description of each of the elements contained in the data are listed below and unless otherwise noted found in the FPDS-NG User's Manual [5]:

**Table:** 

Attribute Name	Attribute Description
Contracting Agency ID	The code for the agency of the contracting office that executed or is otherwise responsible for the transaction
Contracting Agency Name	Specific branch within the DoD requesting contract action⁎⁎
Contracting Office ID	The agency-supplied code of the contracting office that executes the transaction
Contracting Office Name	The agency-supplied name of the contracting office that executes the transaction.
Country Where Award was Issued	Location of execution agent⁎⁎
Major Command Name	Major Command of DoD requesting contracting action
Modification Number	An identifier issued by an agency that uniquely identifies one modification for one contract, agreement, order, etc.
Procurement Instrument Identifier (PIID)	The unique identifier for each contract, agreement, or order. In other words, the individual delivery or task orders (projects)
Referenced IDV PIID	When reporting orders under Indefinite Delivery Vehicles (IDV) such as a Governmentwide Acquisition Contract (GWAC), Indefinite Delivery Contract (IDC), Federal Supply Schedule (FSS), Basic Order Agreement (BOA), or Blanket Purchase Agreement (BPA), report the Procurement Instrument Identifier (Contract Number or Agreement Number) of the IDV. For the initial load of a BPA under an FSS, this is the FSS contract number. Note: BOAs and BPAs are with industry and not with other Federal Agencies. In other words, the parent contract ID of an IDV issued that can have multiple delivery or task orders (PIID) obligated against it.
Referenced IDV Mod Number	When reporting orders under Indefinite Delivery Vehicles (IDV) such as a GWAC, IDC, FSS, BOA, or BPA, report the Modification Number along with Procurement Instrument Identifier (Contract Number or Agreement Number) of the IDV. For the initial load of a BPA under an FSS, this is the FSS contract number. Note: BOAs and BPAs are with industry and not with other Federal Agencies
Transaction Number	Tie Breaker for legal, unique transactions that would otherwise have the same key
Date Signed	The date that a mutually binding agreement was reached. The date signed by the Contracting Officer or the Contractor, whichever is later.
Effective Date	The date that the parties agree will be the starting date for the contract's requirements. The Effective Date cannot be earlier than the Signed Date on the base document.
Completion Date	The [current] completion date of the base contract plus options that have been exercised
Est. Ultimate Completion Date	The estimated or scheduled completion date, including the base contract or order, and all options (if any), whether the options have been exercised or not
Fiscal Year	The fiscal year of action as determined by 'Date Signed'
Funding Agency ID	The agency ID that has provided the preponderance of funding
Funding Agency Name	The agency name that has provided the preponderance of funding (e.g., Dept of the Navy)
Funding Department ID	The Department or Independent Agency ID to which the 'Funding Agency' belongs
Funding Department Name	The Department or Independent Agency name to which the 'Funding Agency' belongs (e.g., DoD)
Funding Office ID	The code provided by the funding agency that identifies the office or other organizational entity that provided the funds for this transaction. If the Funding Agency is DoD, the code must be valid in the DoD Activity Address Code (DODAAC) table. This is a required field when DoD has funded the action.
Funding Office Name	The funding office is the office within the federal agency that is providing the funding for the contract
(Type of IDC)	Identifies whether the IDC or Multi-Agency Contract is Indefinite Delivery/Requirements, Indefinite Delivery/Indefinite Quantity, or Indefinite Delivery/Definite Quantity. A requirements contract provides for filling all actual purchase requirements of designated Government activities for supplies or services during a specified contract period, with deliveries or performance to be scheduled by placing orders with the contractor. A Requirements IDC or Multi-Agency Contract is a contract for all of the agency's requirement for the supplies or services specified, and effective for the period stated, in the IDC or Multi-Agency Contract.
Multiple or Single Award IDV	Indicates whether the contract is one of many that resulted from a single solicitation, all of the contracts are for the same or similar items, and contracting officers are required to compare their requirements with the offerings under more than one contract or are required to acquire the requirement competitively among the awardees
Multi-year Contract Code	A multi-year contract means a contract for the purchase of supplies or services for more than one, but not more than five, program years. Such contracts are issued under specific congressional authority for specific programs. A multi-year contract may provide that performance under the contract during the second and subsequent years of the contract is contingent upon the appropriation of funds, and (if it does so provide) may provide for a cancelation payment to be made to the contractor if appropriations are not made. The key distinguishing difference between multi-year contracts and multiple year contracts is that multi-year contracts buy more than one year of requirement (of a product or service) without establishing and having to exercise an option for each program year after the first
Type of Contract	The type of contract, as defined in FAR Part 16 that applies to this procurement. The following apply to all Awards and IDVs:
	A - Fixed Price Redetermination
	B - Fixed Price Level of Effort
	J - Firm Fixed Price
	K - Fixed Price with Economic Price Adjustment
	L - Fixed Price Incentive
	M - Fixed Price Award Fee
	R - Cost Plus Award Fee
	S - Cost No Fee
	T - Cost Sharing
	U - Cost Plus Fixed Fee
	V - Cost Plus Incentive Fee
	Y - Time and Materials
	Z - Labor Hours
	The following apply to IDVs only:
	1 - Order Dependent (IDV allows pricing arrangement to be determined separately for each order)
	The following apply to Awards only:
	2 - Combination (Applies to Awards where two or more of the above apply)
	3 - Other (Applies to Awards where none of the above apply)
NAICS Code	The North American Industry Classification System (NAICS) codes designate major sectors of the economies of Mexico, Canada, and the United States
NAICS Description	Field providing further information on the description of work in reference to the 'NAICS Code'
Principal Place of Performance State Code	This is the location of the principal plant or place of business where the items will be produced, supplied from stock, or where the service will be performed.
Principal Place of Performance City Name	This is the location of the principal plant or place of business where the items will be produced, supplied from stock, or where the service will be performed.
Principal Place of Performance Country Name	This is the location of the principal plant or place of business where the items will be produced, supplied from stock, or where the service will be performed.
Place of Performance Zip Code	This is the location of the principal plant or place of business where the items will be produced, supplied from stock, or where the service will be performed.
Product or Service Description	A description of the product or service designated by the product code
Product or Service Code	These codes indicate “WHAT” was bought for each contract action reported
Description of Requirement	A brief description of the contract or award
Award or IDV Type	Types of awards:
	- Delivery /Task Order Against IDV
	- Purchase Order
	- Definitive Contract
	- BPA Call
	- Other Transaction Order*
	- Other Transaction Agreement*

	Types of IDVs(Indefinite Delivery Vehicles):
	- Federal Supply Schedule (FSS)
	- Governmentwide Acquisition Contract (GWAC)
	- Basic Ordering Agreement (BOA)
	- Blanket Purchase Agreement (BPA)
	- Indefinite Delivery Contracts (IDC)
	- Other Transaction IDV*
	* Can only be used by DoD, DHS, and HHS
Reason For Modification Description	Reason for modification (change order) which may or may not be applicable:
	A - Additional Work (new agreement, FAR part 6 applies)
	B - Supplemental Agreement for work within scope
	C - Funding Only Action
	D - Change Order
	E - Terminate for Default (complete or partial)
	F - Terminate for Convenience (complete or partial)
	G - Exercise an Option
	H - Definitize Letter Contract
	J - Novation Agreement
	K - Close Out
	L - Definitize Change Order
	M - Other Administrative Action
IDV Type	The type of Indefinite Delivery Vehicle being (IDV) loaded by this transaction. IDV Types include Government-Wide Acquisition Contract (GWAC), Multi-Agency Contract, Other Indefinite Delivery Contract (IDC), Federal Supply Schedule (FSS), Basic Ordering Agreement (BOA), and Blanket Purchase Agreements (BPA)
Extent Competed	A code that represents the competitive nature of the contract:
	A - Full and Open Competition
	B - Not Available for Competition
	C - Not Competed
	D - Full and Open Competition after exclusion of sources
	E - Follow On to Competed Action
	F - Competed under Simplified Acquisitions Program (SAP)
	G - Not Competed under SAP
	CDO - Competitive Delivery Order
	NDO - Non-Competitive Delivery Order
Number of Offers Received	The number of actual offers/bids received in response to the solicitation
Treasury Account Symbol Agency Identifier	Agency Identifier represents the department, agency, or establishment of the U.S. Government that is responsible for the Treasury Account Symbol.
Treasury Account Symbol Main Account Code	The U.S. Federal Agency account code for the agency supplying the preponderance of funding as assigned by the U.S. Treasury ⁎⁎
Treasury Account Symbol Sub Account Code	Identifies a Treasury-defined sub-division of the main account⁎⁎
IDV NAICS Code	The NAICS Code of the parent IDV contract⁎⁎
IDV NAICS Description	The NAICS Description of the parent IDV contract⁎⁎
IDV Contracting Agency ID	The code for the agency of the contracting office that executed the parent IDV contract⁎⁎
IDV Contracting Agency Name	The name of the entity responsible for the initial parent IDV contract action⁎⁎
IDV Department ID	The department ID of the entity responsible for the initial parent IDV contract action⁎⁎
IDV Department Name	The department name of the entity responsible for the initial parent IDV contract action. Typically the U.S. DoD or GSA⁎⁎
IDV Major Program Code	This field is not required, but you may enter it on all IDVs except for an FSS. This is the agency-determined code for a major program within the agency. For an Indefinite Delivery Vehicle, this may be the name of a GWAC (such as ITOPS or COMMITS).
IDV Referenced IDV Agency Code	The agency code that initially input the parent IDV contract⁎⁎
IDV Referenced IDV PIID	The Contract Number of the IDV against which the order is placed
IDV Subcontract Plan	This data element is required for a DCA, Purchase Order, Delivery Order against a BOA, and Part 13 BPA Call. A Delivery Order against FSS, GWAC, and IDC will be propagated. Part 8 BPA Call is Not Applicable. This field indicates whether the contract award required a Subcontracting Plan. This field is also used to provide information to the Electronic Subcontracting Reporting System (eSRS) on awards that have subcontracting plans. Failure to complete this field accurately impacts vendors’ ability to report subcontracting achievement to the eSRS. Select the appropriate value from the drop-down menu. See Data Dictionary Element 11B Use Case for appropriate data entry requirements.

	A - Plan Not Included - No Subcontracting Possibilities
	B - Plan Not Required
	C - Plan Required - Incentive Not Included
	D - Plan Required - Incentive Included
	E - Plan Required (Pre 2004)
	F - Individual Subcontract Plan
	G - Commercial Subcontract Plan
	H - DoD Comprehensive Subcontract Plan
IDV Subcontract Plan Description	A description of the subcontract plan work performed under the parent IDV contract⁎⁎
IDV Type of IDC	This data element is required on an IDC and Populates to the Modification. It is Not Applicable for all other IDVs. This field identifies whether the IDC or Multi-Agency Contract is Indefinite Delivery/Requirements, Indefinite Delivery/Indefinite Quantity, or Indefinite Delivery/Definite Quantity (FAR 16.5). An entry is required for civilian agency and DoD IDCs. Values are listed below:

	A - Indefinite Delivery / Requirements
	B - Indefinite Delivery / Indefinite Quantity
	C - Indefinite Delivery / Definite Quantity
IDV Type of IDC Description	The type of Indefinite Delivery Contract Descriptions of the parent IDV contract⁎⁎
IDV Who Can Use	This data element is required on all IDVs and is Not Applicable for Modifications. This field designates agencies that may place orders against this indefinite delivery vehicle. For the initial award of an IDV, select one of the following:
	− Only My Agency – Only the agency awarding the contract may place orders.
	− All Agencies – All Federal Government agencies may place orders against the contract.
	− Defense – Only Department of Defense agencies may place orders against the contract.
	− Civilian – Only civilian agencies may place orders against the contract.
	− Other – Provide a text statement of which agencies may place orders against the contract.
IDV Who Can Use Description	The description of the Who Can Use field:
	– Only the agency awarding the contract may place orders.
	– All Federal Government agencies may place orders against the contract.
	– Only Department of Defense agencies may place orders against the contract.
	– Only civilian agencies may place orders against the contract.
	– Provide a text statement of which agencies may place orders against the contract.
Base and Exercised Options Value	The contract value for the base contract and any options that have been exercised
Action Obligation	The amount that is obligated or de-obligated by this transaction
Base and All Options Value (Total Contract Value)	Required for all Awards and Modifications except for a BPA Call. It is not required for a Change or Delete/Void. It is the mutually agreed upon total contract or order value including all options (if any). For modifications, this is the change (positive or negative, if any) in the mutually agreed upon total contract value.

⁎⁎ Indicates that the attribute definition was not provided by the FPDS-NG user's manual or wiki, but was provided based on the insight of contracting officers.

## CRediT authorship contribution statement

**Tyler Stout:** Software, Investigation, Validation, Data curation. **Adam Teston:** Investigation, Writing - original draft. **Brent Langhals:** Software, Validation, Data curation, Writing - review & editing. **Justin Delorit:** Writing - original draft, Writing - review & editing. **Carlton Hendrix:** Conceptualization, Supervision, Funding acquisition. **Steven Schuldt:** Writing - review & editing, Supervision, Funding acquisition.

## Declaration of Competing Interest

The Air Force Civil Engineer Center (AFCEC) is the sponsor of this ongoing research effort. The authors declare that they have no known competing financial interests or personal relationships which have, or could be perceived to have, influenced the work reported in this article.

## Appendix Supplementary materials

Image, application 1
